# Re-assessment of selected Baby-Friendly maternity facilities in Accra, Ghana

**DOI:** 10.1186/1746-4358-8-15

**Published:** 2013-11-11

**Authors:** Richmond Nii Okai Aryeetey, Comfort Liousa Antwi

**Affiliations:** 1 School of Public Health, University of Ghana, Legon, P.O. Box LG 13, Accra, Ghana

**Keywords:** Adherence, Baby-Friendly, Breastfeeding, Monitoring, Re-assessment

## Abstract

**Background:**

The Baby-Friendly Hospital Initiative (BFHI) has been implemented in Ghana since 1995. At the end of 2011, about 325 maternity facilities in Ghana had been designated Baby Friendly. However, none had been re-assessed for adherence to the Ten Steps to successful breastfeeding (*Ten Steps*). The current study re-assessed six maternity facilities in Accra for adherence to the *Ten Steps* and the International Code of Marketing of breast milk substitutes (*the Code*).

**Methods:**

Three independent assessors performed the re-assessment using the revised WHO/UNICEF external re-assessment tool (ERT) between April and June, 2011. All sections of the ERT were implemented, except for the HIV/infant feeding section. Assessors interviewed 90 clinical staff of the facilities, 60 pregnant women, and 150 women who had given birth and waiting to be discharged from the hospital. Additionally, observations were completed on neonate feeding and compliance with *the Code*. Data was analyzed to assess adherence to the *Ten Steps* and *the Code*.

**Results:**

In 2010, the six facilities recorded a total of 26,339 deliveries. At discharge, the weighted exclusive breastfeeding rate was 93.8%. None of the facilities adhered completely to the *Ten Steps*. Overall, the rate of adherence to the *Ten Steps* was 42% (range = 30 - 70%). No facility met the criteria for *Steps One* and *Two*. Only *Step Seven* was adhered to by all facilities. Overall compliance with *the Code* was about 54%. Trained staff attrition, high client-staff ratios, inadequate in-service training for new staff, and inadequate support for regional and national program monitoring were identified as barriers to adherence.

**Conclusion:**

Poor adherence to Baby-Friendly practices in designated BFHI facilities was observed in urban Accra. Renewed efforts to support monitoring of designated facilities is recommended.

## Background

Breastfeeding is acknowledged as one of the most important public health interventions for ensuring child survival and development [1,2]. Compared with formula feeding, optimal breastfeeding during early childhood is associated with both short and long term health benefits spanning childhood into adulthood in both developed and developing countries [3,4]. Since the benefits of breastfeeding are dose dependent, exclusively breastfeeding, breastfeeding for a longer duration, or both, is associated with better health and development outcomes. Based on this evidence, breastfeeding exclusively is recommended during the first six months [5]. It is further recommended that from the sixth month, breast milk should be complemented with appropriate and feasible foods, until the end of the second year of life or beyond.

Despite reported prevalent knowledge of the benefits of breast milk, breastfeeding practice is suboptimal in many settings [6]. Globally, exclusive breastfeeding among children below 6 months occurs among only a third of infants [6]. Among children who are breastfed, initiation of breastfeeding is often delayed; only 43% commence breastfeeding within one hour of birth. Misperceptions regarding child feeding, cultural norms, unhealthy marketing and distribution of breast milk substitutes, and inadequate support have been identified as key barriers to optimal breastfeeding practice [7,8].

In 1991, UNICEF and WHO launched the Baby Friendly Hospitals Initiative (BFHI) as a strategy to support, protect, and promote optimal breastfeeding in the health care environment [9]. The initiative recognized breastfeeding as a feeding norm during early childhood and seeks to promote its practice among women who seek delivery care from designated maternity facilities. A key strategy involved designating maternity facilities ‘Baby Friendly’, if they were compliant to a set of breastfeeding best practice indicators known as the ‘*Ten steps* to successful breastfeeding’ (Table 1). In addition, the initiative seeks to promote adherence to the International Code of Marketing of breast milk substitutes (Table 1) popularly referred to as ‘*the code*’ [10]. It has been demonstrated that adherence to the *Ten Steps* provides opportunity for caregivers to become aware of breastfeeding information, learn skills, and receive support needed for optimal breastfeeding and complementary feeding, and thereby improve breastfeeding and other child feeding practices [11,12]. A long-term goal of the initiative is to progressively designate all maternity facilities [13].

**Table 1:** The Ten Steps to successful breastfeeding and the relevant aspects of the code of marketing of breast milk substitutes

	**The Ten Steps**
	*Every facility providing maternity services and care for newborn infants should:*
1	Have a written breastfeeding policy that is routinely communicated to all health care staff.
2	Train all health care staff in skills necessary to implement this policy.
3	Inform all pregnant women about the benefits and management of breastfeeding.
4	Help mothers initiate breastfeeding within half an hour of birth.
5	Show mothers how to breastfeed, and how to maintain lactation even if they should be separated from their infants.
6	Give newborn infants no food or drink other than breast milk, unless medically indicated.
7	Practise rooming-in - that is, allow mothers and infants to remain together - 24 hours a day.
8	Encourage breastfeeding on demand.
9	Give no artificial teats or pacifiers (also called dummies or soothers) to breastfeeding infants.
10	Foster the establishment of breastfeeding support groups and refer mothers to them on discharge from the hospital or clinic.
	**The Code**
	*The following questions must be answered in the affirmative for compliance with the Code:*
1	Does the healthcare facility refuse free or low-cost supplies of breast-milk substitutes, purchasing them for the wholesale price or more?
2	Is all promotion for breast-milk substitutes, bottles, teats, or pacifiers absent from the facility, with no materials displayed or distributed to pregnant women or mothers?
3	Are employees of manufacturers or distributors of breast-milk substitutes, bottles, teats, or pacifiers prohibited from any contact with pregnant women or mothers?
4	Does the hospital refuse free gifts, non-scientific literature, materials or equipment, money or support for in-service education or events from manufacturers or distributors of products within the scope of the Code?

Source: UNICEF [14].

Globally, at the end of 2011, an estimated 21,000 facilities had been certified as ‘Baby-Friendly’, representing about a quarter of all maternity facilities [13]. Generally, rate of designation of facilities has occurred rather slowly in most countries, except among countries in the East Asia and Pacific region. Within sub-regions, there are wide variations in rate of designation. In sub-Saharan Africa, current rate of facility designation is estimated at about 20% [13].

Re-assessment of designated facilities is also recommended every three years [9] to ensure that facilities maintained their adherence to the “*Ten Steps*” and *the Code*. Unfortunately, there is a dearth of information on re-assessment. In Ghana, designation of maternity facilities as ‘Baby-Friendly’ commenced in 1995. At the end of 2010, 325 out of 1527 maternity facilities had been designated Baby-Friendly. While the process of designation required re-assessment of the facilities with respect to the *Ten Steps*, only one designated facility has ever been formally re-assessed (personal communication, National BFHI Program manager). Thus adherence to the *Ten Steps* and *the Code* remains unknown in the designated Baby-Friendly facilities in Ghana.

The current study was designed to re-assess six selected hospitals in the capital of Ghana in order to determine adherence to the *Ten Steps* and *the* C*ode*. The findings presented are intended to inform decisions for further scale up and quality improvement for BFHI in Ghana and elsewhere.

## Methods

Six BFHI designated maternity facilities were included in the study. Data collection followed the protocol outlined in the revised WHO/UNICEF external re-assessment tool (ERT) for carrying out a re-assessment of Baby-Friendly facilities [14]. The assessment was carried out by accredited national assessors of the BFHI program by the School of Public Health at the request of the Ghana Health Service. The facilities assessed were all public-managed, and included one referral hospital, four primary hospitals and one community health center. All facilities were located in the urban Accra Metropolis, which is the capital city of Ghana. Data collection for the study was carried out between April and June 2011.

The number of staff and clients who were included in the study is indicated in (Table 2). At each facility, the assessors collected data using in-depth interviews, structured interviews, and observation of the facility premises, as well as observation of practices by staff and maternity clients relating to *the Code* and the *Ten Steps*. The in-depth interviews were conducted with the facility administrator to learn about maternity and postnatal services provided by the facility and the capacity available to deliver the services. In-depth interviews were conducted with the staff in order to identify the reasons for failing to adhere to the *Ten Steps* and *the Code*. Client in-depth interviews explored barriers relating to practicing appropriate breastfeeding initiation and maintenance and support received from clinical staff. Clients were also observed in order to determine their skills and capacity for applying optimal feeding practices relating to the *Ten Steps*. Relevant facility documentation on deliveries and antenatal care were also reviewed to provide contextual information about each facility [14].

**Table 2:** Number and category of respondents included in the facility re-assessment

**Respondents**	**Maternity facilities**					
	**A**	**B**	**C**	**D**	**E**	**F**
Non-clinical hospital staff	1	1	1	1	1	1
Clinical staff	15	15	15	15	15	15
Pregnant women	10	10	10	10	10	10
Postnatal women: normal delivery	15	15	15	15	15	15
Postnatal women: child in intensive care	10	10	10	10	10	- *
Total	51	51	51	51	51	41

*Facility does not have neonatal intensive care unit.

Facility names have been replaced with alphabet letters to conceal their identity.

Furthermore, structured interviews were completed with facility staff including administrators, Principal nursing officers, midwives, and other nurses working across various units of the facility i.e. antenatal, labour, and postnatal units, to assess their knowledge and skills regarding *the Code* and the *Ten Steps*. Observations of antenatal, labour/delivery, postpartum, child welfare, and special care baby care units (where available) were carried out to document display of facility breastfeeding policy as well as Baby-Friendly practices of both staff and clients in the facility.

The items in the revised UNICEF ERT for Baby-Friendly Hospitals were used for the re-assessment [9,14]. All, sections of the tool were administered, except the optional questions on HIV and infant feeding, and mother-friendly care. The study protocol and tools were reviewed and approved by the Ghana Health Service’ Ethical Review Committee (GHS-ERC:51/2/11). For the purpose of confidentiality, the facilities are represented with alphabet letters.

### Analysis of data

Analysis was carried out manually utilizing the summary sheets accompanying the revised UNICEF ERT. Observations and interview responses were used to complete the performance indicators of *the Code* and the *Ten Steps* for each facility. Each *Step* or *Code* had a minimum number of indicator that were scored as a percentage and served as performance indicator. To adhere to a particular step, an institution must score the minimum percentage threshold or higher on the total number of indicators required for that step, otherwise, it fails. Adherence to the *Ten Steps* was computed as a ratio of the number of steps adhered to by the institution to total number of steps (that is 10). In the case of *the Code*, the adherence was a ratio with four as the denominator since there were four indicators [14].

## Results

The facilities included in the re-assessment had all been designated Baby-Friendly earlier than year 2005. The earliest among them to be designated Baby-Friendly was facility C in 1996. Thereafter, facilities A and D were designated in 2001; E and F in 2003, and B in 2004. Across the six facilities, a total of 296 respondents were interviewed including 6 facility administrators, 90 clinical staff and 200 women seeking antenatal and postnatal care. Altogether, the facilities delivered a total of 26,339 children in the previous year. The proportion of children delivered in the previous year who were being exclusively breastfed at discharge ranged between 87% and 100% across the facilities. All facilities had capacity to provide complete emergency obstetric services, except Facility F.

Table 3 shows a generally poor adherence of the facilities to the *Ten Steps* to successful breastfeeding. Total adherence rate was 42% across the six facilities, ranging from 30% in the lowest to 70% for the highest. Facility E was the best performing facility, adhering to seven out of the 10 steps. Three facilities, including Facility A which is a major referral hospital, only met three of the *10 Steps*. None of the facilities met the criteria for *Steps One* and *Two*. Only *Step* seven was adhered to by all the facilities. *Steps Six* and *Nine* were adhered to by all facilities, except one. Table 3 further shows that most facilities missed almost all the indicators used for evaluating *Steps One* through *Five*.

**Table 3:** Compliance with the Ten Steps to successful breastfeeding in selected Baby-Friendly facilities in Accra, Ghana

**Steps to successful breastfeeding**	**Maternity facilities**						**Total compliance by steps, %**
	**A**	**B**	**C**	**D**	**E**	**F**	
1							0
2							0
3		√			√		33
4					√		17
5					√		17
6	√	√	√	√	√		83
7	√	√	√	√	√	√	100
8		√					17
9	√	√		√	√	√	83
10		√	√		√	√	67
Total compliance by facilities, %	30	60	30	30	70	30	42

√ minimum performance indicator for the step was achieved.

Facility names have been replaced with alphabets to conceal their identify.

A similar situation of poor adherence was observed for *the Code* on marketing of breast milk substitutes (Table 4). Only one facility (E) was able to achieve all the indicators for which it was assessed. Also, only one facility (E) met the indicator requiring staff to know reasons for disallowing manufacturing companies to distribute free formula samples to companies. On the other hand, observation showed that all facilities kept formula cans and prepared bottles out of view. Table 5 shows that most facilities missed key indicators used for evaluating *the Code*. With the exception of facilities B and E, all the other facilities failed to comply with almost all *the Code* indicators. The overall adherence rate for *the Code* was 54%.

**Table 4:** Number of compliance indicators that were missed by each facility re-assessed

**Compliance criteria (the step and the code)**	**Minimum number of indicators needed for compliance**^**a**^	**Number of indicators missed per facility**					
		**A**	**B**	**C**	**D**	**E**	**F**
1	5	4	4	3	5	2	4
2	9	9	7	8	9	7	9
3	2	2	0	2	2	0	2
4	3	3	3	3	3	0	3
5	4	4	2	4	3	2	4
6	5	0	0	1	2	1	2
7	2	0	0	1	0	0	0
8	2	2	0	2	2	2	2
9	3	0	0	0	0	0	0
10	1	1	0	1	1	0	0
Code	4	3	2	2	2	0	2
Total	39	28	18	27	29	14	28

^a^The criteria on counseling for HIV + mothers was not applied, thus 5 out of the 6 criteria were utilized for assessment.

Facility names have been replaced with alphabet letters to conceal their identity.

**Table 5:** Compliance with the international code on marketing of breast milk substitutes in selected Baby-Friendly facilities in Accra, Ghana

**Indicators of compliance**	**Maternity facilities**						**Total compliance by indicator, %**
	**A**	**B**	**C**	**D**	**E**	**F**	
Facility purchases infant formula at wholesale price or more							-
No BMS, Bottles, teats, or pacifiers displayed for promotional purposes		√	√	√	√	√	83
BMS cans and prepared bottles are kept out of view	√	√	√	√	√	√	100
Clinical staff knowledge on why giving free formula samples to mothers is not good					√		17
Total compliance by facility, %	25	50	50	50	100	33	54.5

BMS = Breast milk substitutes.

√ Minimum performance for the code indicator was achieved.

Facility names have been replaced with alphabet letters to conceal their identity.

## Discussion

A rationale for conducting the re-assessments was to determine whether the training and institutional re-orientation needed to remain designated as Baby-Friendly was being sustained and that it translated into improved practices. We expected that the findings from the assessment in the Accra Metropolis would serve as a gauge for BFHI performance elsewhere in Ghana, where the facilities are less endowed with often less skilled capacity. Our finding that none of the six designated facilities in the Accra Metropolis was adherent to the *Ten Steps* to successful breastfeeding was surprising. It suggested that the adherence to the T*en Steps* in designated facilities outside Accra may be even poorer than observed in Accra. In addition to these six facilities which have performed poorly on the *Ten Steps,* all the other designated maternity facilities which have not been re-assessed continue to be advertised as Baby-Friendly with potential adverse implications for infant breastfeeding promotion; elsewhere, the designation will have been lost until they meet the BFHI standards [15,16].

None of the facilities was compliant with *Steps One* and *Two*. This was despite the fact that a written policy on the BFHI existed in all the facilities. The deficiency, however was that the existing policies did not communicate all the *Ten Steps* to successful breastfeeding, except in one facility. Also, in four of the facilities, although the policy was available, it was not displayed at relevant places for the benefit of staff and clients. Concerning *Step* Two, lack of funds was given by the hospital management as the reason for failing to utilize the existing training guide to build the capacity of the clinical staff on *the Code* and the *Ten Steps*. This excuse may be because in Ghana, it is not uncommon for staff working in public institutions to receive training outside the workplace setting and also to earn extra income (often paid by external organizations providing the training) for participating in a training program. Thus staff are less motivated to participate in training that is not linked with extra income.

There was also low adherence with *Steps Three, Four, Five* and *Eight*. This is not surprising since the clinical staff were not adequately prepared through training to support mothers to practice appropriate feeding in the immediate postpartum period. Poor knowledge and skills of clinical staff to support women in breastfeeding is not a surprising finding as reported by Okolo and Ogbonna in Nigeria [17]. Also, the finding that *Step Seven* was adhered to by all facilities is aided by space constraints in most hospitals, thus facilitating the keeping of infants with their mothers.

A similar pattern was observed for adherence to *the Code* on marketing of breast milk substitutes. Our findings show that there was low adherence with *the Code* in the six maternity facilities. Even more disturbing is the finding that the health staff had poor knowledge on the reasons for not permitting distribution of free formula samples. Two key implications are drawn from this finding of poor adherence to the BFHI standards. Firstly, many mothers who deliver their babies at designated Baby-Friendly facilities may be missing the opportunity to receive adequate support to breastfeed appropriately. Secondly, the low adherence may partly explain the low rates of timely breastfeeding initiation in Ghana [18,19].

The literature on BFHI has only a few published reports on facility re-assessments. In Brazil, Maura de Araujo and Schmitz [20] have reported that out of 172 designated hospitals reassessed, 82% were fully compliant to all the steps. In their study, the steps that had the lowest compliance were steps two and three, bearing similarity to our findings. Earlier in 1999, Dodgson et al., had reported low compliance with the *Ten Steps* in 95 Minnesota Hospitals in the early stages of implementing BFHI [21]. In the African context, however, no published reports on BFHI re-assessments were identified in our search of the literature. Perhaps, this is the first reported study on re-assessment of BFHI in the sub-Saharan Africa region.

The facility staff attributed the high rate of non-adherence to both the *Ten Steps* and *the Code* to multiple reasons including high trained staff attrition, inadequate in-service training for new staff, high client-staff ratios, and inadequate support for regional and national program monitoring. Beyond these barriers, there is also lack of strong leadership and sustained program planning to allow integration of BFHI into existing health system structures. As a result, only a few trained national assessors have the mandate to go round the entire nation to perform supportive monitoring as well as assessments and training for designation. Since the assessors carry out these activities in addition to their primary duties, both facility designation and monitoring is often left unattended to. In the future, the Ghana Health services and the Ministry of health should consider decentralizing the assessment procedure in order to remove bottlenecks in mobilizing assessors across the country.

It is important to recognize that re-assessment of designated Baby-Friendly facilities is recommended at least every three years [9]. In Ghana, however, re-assessment has not been implemented routinely. Rather, there is periodic supportive monitoring that is carried out by a National team of trained assessors. The frequency of supportive monitoring is, however, infrequent due to inadequate funding of the BFHI program. As a result, only a few of the designated facilities benefit from monitoring visits by the assessors each year.

The findings of this study should be interpreted also within the context that the re-assessment tools utilized were the revised versions of the tools that were used to designate the facilities. There are key differences that have been identified between the original assessment tools and the revised version. It is possible, therefore, that if the original tools had been used, different levels of compliance may have been observed. Nevertheless, the findings based on the revised tools reflect current expectations of the BFHI program and should serve as basis for interpreting future re-assessments.

## Conclusions

The current study found that six maternity facilities designated as Baby-Friendly in Metropolitan Accra were only partially adherent (42%) to the *Ten Steps* to successful breastfeeding. Indeed none of the facilities scored a 100% adherence to the BFHI standards. Adherence across facilities ranged between 30 and 70% with four out of six facilities having adherence rate of 30%. Adherence across the steps ranged between zero and 100%; most of the steps (60%) ranged between zero and 33%. There was also low adherence for the code on marketing of breast milk substitutes. To improve adherence, there is a need for the health system to strengthen capacity at the regional and district levels for re-assessment and monitoring. In addition, interventions to improve clinical staff capacity will be essential for improving compliance with BFHI performance at the facility level.

## Competing interests

Both authors declare that they have no competing interests.

## Authors’ contributions

RNOA conceived the study, participated in study design, supervised study tools development, as well as analysis and preparation of the manuscript. CLA, participated in study design, data collection, data analysis and revising the manuscript. Both authors read and approved the final manuscript.
